# Evolutionary Accessibility of Mutational Pathways

**DOI:** 10.1371/journal.pcbi.1002134

**Published:** 2011-08-18

**Authors:** Jasper Franke, Alexander Klözer, J. Arjan G. M. de Visser, Joachim Krug

**Affiliations:** 1 Institute of Theoretical Physics, University of Cologne, Köln, Germany; 2 Laboratory of Genetics, Wageningen University, Wageningen, Netherlands; University of Texas at Austin, United States of America

## Abstract

Functional effects of different mutations are known to combine to the total effect in highly nontrivial ways. For the trait under evolutionary selection (‘fitness’), measured values over all possible combinations of a set of mutations yield a fitness landscape that determines which mutational states can be reached from a given initial genotype. Understanding the accessibility properties of fitness landscapes is conceptually important in answering questions about the predictability and repeatability of evolutionary adaptation. Here we theoretically investigate accessibility of the globally optimal state on a wide variety of model landscapes, including landscapes with tunable ruggedness as well as neutral ‘holey’ landscapes. We define a mutational pathway to be accessible if it contains the minimal number of mutations required to reach the target genotype, and if fitness increases in each mutational step. Under this definition accessibility is high, in the sense that at least one accessible pathway exists with a substantial probability that approaches unity as the dimensionality of the fitness landscape (set by the number of mutational loci) becomes large. At the same time the number of alternative accessible pathways grows without bounds. We test the model predictions against an empirical 8-locus fitness landscape obtained for the filamentous fungus *Aspergillus niger*. By analyzing subgraphs of the full landscape containing different subsets of mutations, we are able to probe the mutational distance scale in the empirical data. The predicted effect of high accessibility is supported by the empirical data and is very robust, which we argue reflects the generic topology of sequence spaces. Together with the restrictive assumptions that lie in our definition of accessibility, this implies that the globally optimal configuration should be accessible to genome wide evolution, but the repeatability of evolutionary trajectories is limited owing to the presence of a large number of alternative mutational pathways.

## Author Summary

Fitness landscapes describe the fitness of related genotypes in a given environment, and can be used to identify which mutational steps lead towards higher fitness under particular evolutionary scenarios. The structure of a fitness landscape results from the way mutations interact in determining fitness, and can be smooth when mutations have multiplicative effect or rugged when interactions are strong and of opposite sign. Little is known about the structure of real fitness landscapes. Here, we study the evolutionary accessibility of fitness landscapes by using various landscape models with tunable ruggedness, and compare the results with an empirical fitness landscape involving eight marker mutations in the fungus *Aspergillus niger*. We ask how many mutational pathways from a low-fitness to the globally optimal genotype are accessible by natural selection in the sense that each step increases fitness. We find that for all landscapes with lower than maximal ruggedness the number of accessible pathways increases with increases of the number of loci involved, despite decreases in the accessibility for each pathway individually. We also find that models with intermediate ruggedness describe the *A. niger* data best.

## Introduction

Mutations are the main sources of evolutionary novelty, and as such constitute a key driving force in evolution. They act on the genetic constitution of an organism at very different levels, from single nucleotide substitutions to large-scale chromosomal modifications. Selection, a second major evolutionary force, favors organisms best adapted to their respective surroundings. Selection acts on the fitness of the organism. How fitness is connected to specific traits such as reproduction or survival depends strongly on the environmental conditions, but indirectly it can be viewed as a function of the organism's genotype.

If one considers mutations at more than one locus, it is not at all clear how they combine in their final effect on fitness. Two mutations that individually have no significant effect on a trait under selection can in combination be highly advantageous or deleterious. Well known examples for such epistatic interactions [1] include resistance evolution in pathogens [2]–[4] or metabolic changes in yeast [5]. In general, the presence of epistatic interactions makes the fitness landscape more rugged, particularly when epistasis affects the sign of the fitness effects of mutations [6]–[8]. Fitness landscapes are most easily dealt with in the context of asexual haploid organisms, and we will restrict our considerations here to this case.

In a remarkable recent development, several experimental studies have probed the effect of epistatic interactions on fitness landscapes [3], [4], [7], [9]–[16]. Most of these studies are based on two genotypes, one that is well adapted to the given environment, and another that differs by a known, small set of mutations; the largest landscapes studied so far involve five mutations [3], [10], [16]. All (or some fraction of the) intermediate genotypes are then constructed and their fitness measured. However, selection in natural populations does not act on small, carefully selected sets of mutations, but rather on all possible beneficial mutations that occur anywhere in the genome, making the number of possible mutations many orders of magnitude greater than those considered in empirical studies.

**Figure 1:**
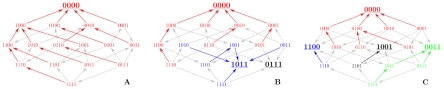
Graphical representation of three fitness landscapes of size 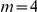 extracted from the empirical 8-locus fitness data set for *A. niger*. The presence/absence of a given mutation is indicated by 1/0. Arrows point towards higher fitness, local maxima are enlarged and underlined, and colors mark basins of attraction of maxima under a greedy (steepest ascent) adaptive walk. (A) All combinations of mutations *argH12*, *pyrA5*, *leuA1*, *oliC2*. This landscape has a single fitness maximum (the wildtype), but only 9 out of 4! = 24 paths from {1111} to {0000} are accessible. (B) Mutations *argH12*, *pyrA5*, *leuA1*, *pheA1*. This landscape has three maxima and no accessible path. (C) Mutations *fwnA1*, *leuA1*, *oliC2*, *crnB12*. The landscape has four maxima and 2 accessible paths.

Figure 1 shows three sample landscapes obtained from an empirical 8-locus data set of fitness values for the fungus *Aspergillus niger* originally obtained in [17] (see **Materials and Methods** for details on the data set and its representations). These landscapes display a wide variation in topography, and despite their moderate size of 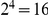 genotypes, the combinatorial proliferation of possible mutational pathways makes it difficult to infer the adaptive fate of a population without explicit simulation [10]. In fact, in view of the broad range of possible landscape topographies, even a thorough understanding of evolution on one of these landscapes would be of limited use when confronted with another subset of mutations or even fitness landscapes from a different organism. Instead, one would like to understand and quantify the typical features of *ensembles* of fitness landscape, where an ensemble can be formed e.g. by selecting different subsets of mutations from an empirical data set, or by generating different realizations of a random landscape model.

Although genome-wide surveys of pairwise epistatic interactions have recently become feasible [18], exploring an entire fitness landscape on a genome-wide scale remains an elusive goal. In this situation theoretical considerations are indispensable to assess the influence of epistasis on the outcome of evolutionary adaptation. Here, we aim to perform part of this task by answering the following question: Does epistasis make the global fitness optimum selectively inaccessible?

This question has a long history in evolutionary theory, and two contradictory intuitions can be discerned in the still ongoing debate [1]. One viewpoint generally attributed to Fisher [19] emphasizes the proliferation of mutational pathways in high dimensional genotype spaces to argue that, because of the sheer number of possible paths, accessibility will remain high. The second line of argument originally formulated by Wright [20], and more recently promoted by Kauffman [21] and others, focuses instead on the proliferation of local fitness maxima, which present obstacles to adaptation and reduce accessibility with increasing genotypic dimensionality. Here we show that both views are valid at a qualitative level, but that Fisher's scenario prevails on the basis of a specific, quantitative definition of accessibility, since the number of accessible pathways grows much faster with landscape dimensionality than the inaccessibility per pathway as long as the fitness landscape is not completely uncorrelated. Moreover, our analysis of accessibility in the empirical *A. niger* data set illustrated in Figure 1 shows how evolutionary accessibility can be used to quantify the degree of sign epistasis in a given fitness landscape.

### Mathematical framework

The dynamics of adaptation of a haploid asexual population on a given fitness landscape is governed by population size 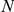, selection strength 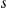 and mutation rate 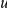, and different regimes for these parameters have been identified [22]–[24]. Here we assume a ‘strong-selection/weak mutation’ (SSWM) regime [25], [26], which implies that mutations are selected one by one and prohibits the populations from crossing valleys of fitness. In natural populations of sufficient size, a number of double mutants is present at all times, and the crossing of fitness valleys can be relatively facile [27], [28]; the SSWM assumption may therefore seem overly restrictive. However, we will see that even under these conditions, the landscapes considered are typically very accessible.

In the remainder of the paper, the genetic configuration of the organism will be represented as a binary sequence 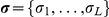 of total length 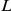, where 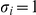 (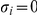) stands for the presence (absence) of a given mutation in the landscape of interest. The SSWM assumption together with the fact that we only consider binary sequences gives the configuration space the topological structure of a hypercube of dimension 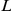. Accessibility can then be quantified by studying the *accessible mutational paths* [2], [3], [29]. A mutational path is a collection of point mutations connecting an initial state 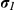 with a final state 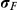. If these two states differ at 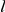 sites, there are 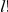 shortest paths connecting them, corresponding to the different orders in which the mutations can be introduced into the population [30]. The assumed weak mutation rate implies that paths longer than the shortest possible path have a much lower probability of occurrence and hence are not considered here, adding to the constraints already imposed on accessibility. A mutational path is considered selectively accessible (or accessible for short) if the fitness values encountered along it are monotonically increasing; thus along such a path, the population never encounters a decline in fitness. If two states are separated by a fitness valley, the path is inaccessible. Neutral mutations are generally not detected in the empirical fitness data sets of interest here, though they may be present at a finer scale of resolution [31]. In our modeling we therefore assume that the fitness values of neighboring genotypes can always be distinguished (but see the discussion of the holey landscape model below).

Unlike Ref. [3] we only consider whether a given path is at all accessible or not, independent of the probability of the path actually being found by the population. Our reason for focusing on this restricted notion of accessibility is that it can be formulated solely with reference to the underlying fitness landscape, without the need to specify the adaptive dynamics of the population (see also **Discussion**). The endpoint of the paths considered here, much like in the experimental studies [3], [4], [10], is the global fitness maximum, and the starting point is the ‘antipodal’ sequence which differs from the optimal sequence at all 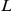 loci. Because it is at the opposite end of the configuration space, these are the longest direct paths. As such, they are *a priori* the least likely to be accessible and thus give a lower limit on the accessibility of typical paths (note that the mean length of the path from a randomly chosen genotype to the global maximum is 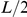).

**Figure 2:**
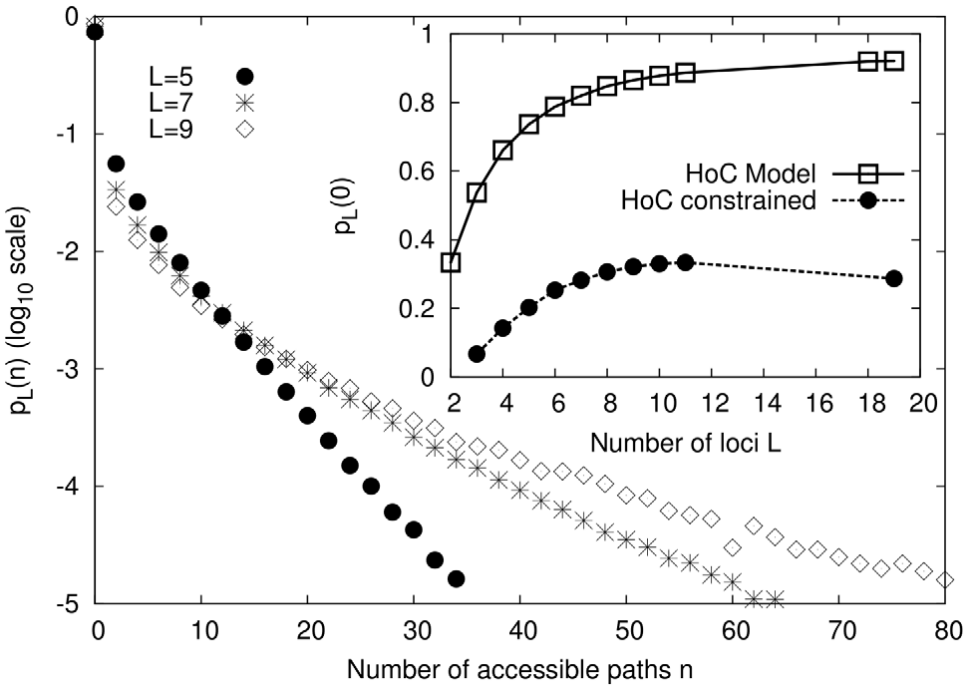
Accessibility of mutational pathways in the House-of-Cards model. Main figure shows the distribution of the number of accessible paths for three different sequence lengths in the HoC model in semi-logarithmic scales. The value of 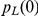 is an outlier, indicating that a large fraction of landscapes have no accessible paths at all. This is a typical feature of rugged fitness landscapes of moderate dimensionality 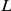, see Figures S4 and S5. Inset shows 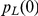 as function of 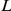 for the HoC model. The top curve makes no assumptions about the antipodal sequence, while the bottom curve assumes it to be the global fitness minimum. Note the decline in the bottom curve.

For a fitness landscape comprised of up to 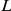 mutations, there are a total of 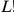 paths connecting the antipodal sequence to the global maximum. How many of them are selectively accessible in the sense described above? Given that natural selection is expected to act genome-wide, we are interested in the behavior of accessibility properties when the number of loci 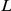 becomes very large. Two questions are of particular interest: What is the probability of finding at least one accessible path, and what number of accessible paths can one expect to find on average? The first question addresses the overall accessibility of the global fitness maximum [32], while the second question is relevant for the repeatability of evolution: If there are many possible mutational pathways connecting the initial genotype to the global maximum, depending on population dynamics different pathways can be chosen in replicate experiments and repeatability will be low. To address these questions in a quantitative way, consider a sample of fitness landscapes, obtained e.g. as random realizations of a landscape model or by choosing subsets of mutations from a large empirical data set (see Figure 1). The fraction of these that have exactly 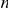 accessible paths is denoted by 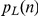, and gives an estimate of the probability that a given fitness landscape has 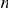 accessible paths (cf. Figure 2). The expected number of paths is given by the mean of this probability distribution,
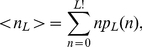
(1)and 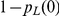 is the probability to find at least one accessible path. The behavior of these two quantities will be investigated in the following, both for model landscapes and on the basis of empirical data.

## Results

### House of Cards (HoC) model

**Figure 3:**
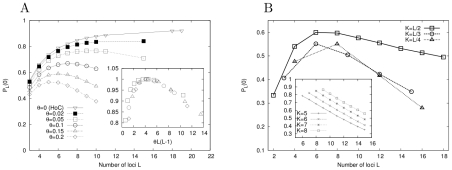
Accessibility in fitness landscape models with tunable ruggedness. (A) Behavior of 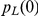 in the RMF model as function of the correlation parameter 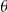. Inset shows normalized rescaled curves, all taking their maximum at 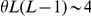. This implies that 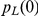 increases monotonically only for 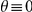. (B) Probability 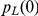 for the 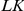 model as a function of 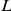 at fixed 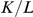 (main figure) and fixed 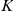 (inset), respectively.

Consider a model where fitness values are uncorrelated and a single mutation may change fitness completely [21], [24], [29]; following Kingman [33] we refer to this as the ‘House of Cards’ model. In real organisms one expects fitnesses of closely related genotypes to be at least somewhat correlated, and in this sense the HoC model serves as a null model. The expected number of accessible paths can be computed exactly by a simple order statistics argument [34]. Each of the 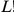 shortest paths contains 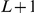 genotypes. Out of the 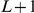 fitness values encountered along a path, all but the last one (which is known to be the global maximum) are arranged in any order with equal probability. One of the 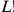 possible orderings is monotonic in fitness, hence for the HoC model
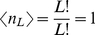
(2)for all 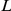. The probability 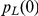 of not finding any path is more difficult to compute and was so far only analyzed by numerical simulations. We find that for sequence lengths up to 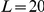, 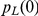 appears to approach unity, see inset of Figure 2 and Figure S1. Whether this is asymptotically true remains to be established, but the scaling plot in the inset of Figure 3 suggests that 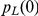 is indeed monotonically increasing for all finite 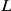.

This behavior changes drastically when the antipodal state is required to be the global fitness minimum. This case was considered previously by Carneiro and Hartl [32], who postulated that 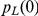 saturates to an asymptotic value around 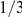 for large 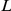. However by continuing the simulations to 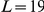, one sees a clear decline (inset of Figure 2), indicating that accessibility *increases* with increasing 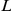. We will see in the following that this is in fact the generic situation.

### Rough Mount Fuji (RMF) model

Next we ask what happens if some fitness correlations are introduced. The Rough Mount Fuji (RMF) model [35] accomplishes just that: Denoting the number of mutations separating a given genotype 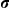 from the global optimum by 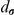, the RMF model assigns fitness values according to
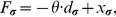
(3)where 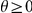 is a constant and the 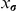 are independent normal random variables with zero mean and unit variance. When 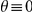 the RMF reduces to the HoC case, and thus it can serve as starting point for approximate calculations to first order in 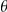. For the expected number of accessible paths one obtains [34]

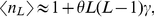
(4)where 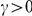 and terms of higher order have been neglected (see also Eq. (7)). In this limit 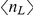 grows like 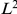 for large 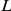 and constant 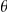. Compared to the HoC case 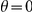, this shows that the large 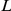-behavior of a landscape with even the slightest correlation between fitness values is substantially different from the case without correlations.

The probability of finding no accessible paths was again obtained by numerical simulation, and is shown in Figure 3(A). In striking contrast to the unconstrained HoC model, the probability 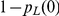 of finding at least one accessible path is seen to increase for large 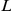. Motivated by the result (4), in the inset of Figure 3(A) the simulation results are plotted as a function of 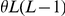, which leads to an approximate collapse of the different data sets. On the basis of these results we conjecture that, for any 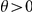, the probability 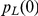 decreases for large 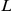, and most likely approaches zero asymptotically for 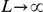.

### LK model

Better known as the NK-model [21], [36], this classical model explicitly takes into account epistatic interactions among different loci. Each of the 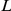 sites in the genome is assigned a certain number 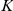 of other sites with which it interacts, and for each of the possible 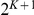 states of this set of interacting loci the site under consideration contributes to the fitness by a random amount. Thus the parameter 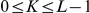 defines the size of the epistatically interacting parts of the sequence and provides a measure for the amount of epistasis. Like the RMF model, the 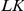 model reduces in one limit to the HoC case, which is realized for 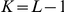.

Due to the construction of the model, even local properties such as the number of local fitness optima [37], [38] are generally very difficult to compute. Figure 3(B) shows the variation of 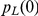 with 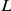 obtained from numerical simulations of the 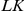 model. In this figure two different relations between 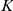 (the number of interacting loci) and 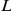 (the total number of loci) were employed. In the main plot the fraction of interacting loci 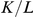 was kept constant. Under this scenario, the curves show a non-monotonic behavior of 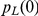 similar to that of the RMF model at constant epistasis parameter 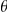. In the inset, the number of interacting loci 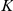 is kept fixed, which results in a monotonic decrease of 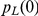. A third possibility is to fix the *difference* 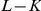 (the number of non-interacting loci), see Figure S2. In this case one can argue that for 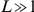, the difference in behavior between 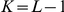 and 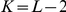, say, should not be substantial, and indeed the curves for 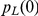 seem to be monotonically increasing with 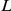, showing qualitatively the same behavior as the curve for 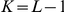, which is equivalent to the HoC model. Finally, in Figure S3 we show the expected number of accessible paths for different values of 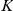 and 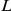. The data are seen to interpolate smoothly between the known limits 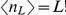 for 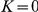 and 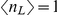 for 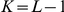.

### Holey landscapes

The neutral theory of evolution [39] implies a very simple, flat fitness landscape without maxima or minima. When strongly deleterious mutations are included, the resulting fitness landscape has plateaus of viable states and stretches of lethal states [40]. Such ‘holey’ landscapes can be mapped [41] to the problem of percolation, a paradigm of statistical physics [42]. In percolation, each configuration is either viable (fitness 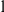) with probability 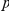 or lethal (fitness 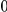) with probability 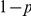, independent of the others. Our definition of accessibility must be adapted in this case, as there is no notion of increasing fitness and no global fitness optimum. However, one can still ask the question whether it is possible to get from one end of configuration space to the other on a shortest path of length 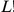 without encountering a ‘hole’, i.e. a non-viable state. Apart from the restriction to shortest paths, the probability 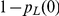 of finding at least one connecting path then corresponds to the percolation probability.

The percolation problem on the hypercube differs from the standard case of percolation on finite-dimensional lattices [42] in that the parameter 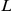 represents both the dimensionality and the diameter of the configuration space. Percolation properties are therefore described by statements that hold asymptotically for large 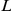 under some suitable scaling of the viability probability 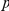 [43], [44]. Specifically, when 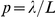 for some constant 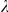, it is known that for 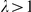 a giant connected set of viable genotypes emerges for 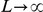. Conversely, taking 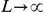 at fixed 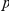 one expects that two antipodal genotypes are connected by a path with a probability approaching unity. Indeed, the simulation results shown in Figure S4 support the conjecture that the quantity corresponding to 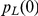 vanishes for large 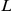 and any 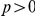. The equivalent of computing 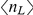 is straightforward: The probability that 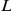 consecutive states are viable factorizes by independence of the fitness values to the product of the individual probabilities of viability, to simply yield 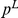, which, as 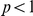, decays exponentially. We already know that there are 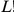 possible paths in the sequence space, thus we find
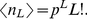
(5)Since 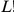 grows faster than 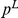 declines, 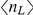 grows without bounds for large 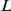.

### Comparison to empirical data

Next we compare the predictions of the models described so far to the results of the analysis of a large empirical data set obtained from fitness measurements for the asexual filamentous fungus *A. niger*. As described in more detail in **Materials and Methods**, we analyzed the accessibility properties of ensembles of subgraphs containing subsets of 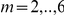 out of a total of 8 mutations which are individually deleterious but display significant epistatic interactions [17]. The full data set contains fitness values for 186 out of the 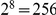 possible strains, and statistical analysis shows that the 70 missing combinations can be treated as non-viable genotypes with zero fitness. The distribution of the non-viable genotypes in the subgraph ensemble is well described by a simple two-parameter model which reveals that the lysine deficiency mutation *lysD25* is about 25 times more likely to cause lethality than the other seven mutations (see **Materials and Methods**).

**Figure 4:**
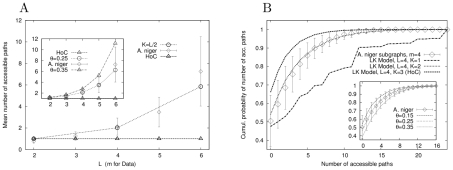
Comparison of models to empirical data. (A) Mean number of accessible paths for HoC, RMF and 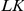 models compared to the empirical *A. niger* data. With the exception of the HoC model, all curves show an increase of 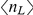 with 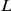. Both RMF (inset) and 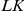 (main plot) models can be fit to the empirical data. Error bars on the empirical data represent standard deviations obtained from the resampling analysis. (B) Cumulative probability of the number of accessible paths as observed in the empirical fitness landscape compared to 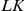 (main plot) and RMF (inset) model. Error bars represent the standard deviation estimated by the resampling method.

Results of the subgraph analysis are displayed in Table 1 and in Figure 4. The data in Figure 4(A) show a systematic increase of the average number of accessible paths with the mutational distance 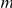 in the empirical data, which rules out the null hypothesis of uncorrelated fitness values and is quantitatively consistent with the RMF model with 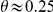 (inset). The data for even subgraph sizes 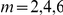 are equally well described by the 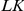 model with 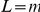 and 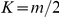 (main figure). Alternatively, the empirical data can be compared to the results of a subgraph analysis of a 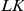 fitness landscape with fixed 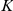 and 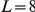 (Figure S5). While the fit between model and data is less satisfactory than that shown in Figure 4(A), the comparison is consistent with a value of 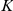 between 4 and 5, which again indicates that each locus interacts with roughly half of the other loci.

**Table 1:** Subgraphs of the *A. niger* data set.

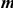	# SG	# VSG	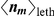	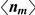	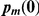
2	28	20 (19.5)	1.61 (1.72)	0.82	0.36
3	56	29 (28.1)	4.05 (4.22)	1.34	0.39
4	70	19 (19.5)	12.53 (13.19)	2.01	0.50
5	56	4 (4.9)	55.32 (48.81)	3.16	0.63
6	28	0 (0.2)	246.0 (201.16)	6.07	0.68

The table summarizes properties of subgraphs of sizes 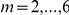 of the empirical *A. niger* fitness landscape. Second column shows the total number of subgraphs 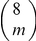 and third column the number of viable subgraphs not containing any non-viable genotypes, with the model prediction (10) given in brackets. Fourth column contains the number of accessible paths that would be present if accessibility were reduced only because of the presence of non-viable genotype, with the model prediction (11) shown in brackets. Finally, the last two columns show the mean number of accessible paths 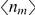 and the probability of no accessible path 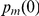, respectively, computed from the full subgraph ensemble.

Further analysis of statistical properties of the *A. niger* landscape confirms this conclusion. As an example, in Figure 4(B) we display the cumulative distribution of the number of accessible paths
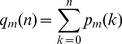
(6)obtained from the analysis of the largest subgraph ensemble with 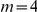. The main figure shows that good quantitative agreement is achieved with the 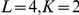 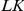 model. The inset displays a similar comparison to the RMF-model, which leads to the estimate 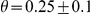 for the roughness parameter, in close agreement with the estimate obtained from 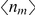.

For the 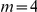 subgraph ensemble, the probability 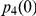 of finding no accessible path is approximately 0.5. Corresponding estimates 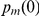 for other values of 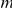 can be found in the last column of Table 1. Up to 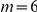, the probability is found to increase with 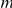, which implies that the ultimate increase of accessibility (decrease in 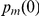) predicted by the models cannot yet be seen on the scale of the empirical data. This is consistent with the estimates of the epistasis parameters 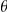 and 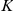 mentioned above, for which the maximum in 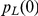 is reached at or beyond six loci (compare to Figure 3).

## Discussion

### Evolutionary accessibility

The models considered here represent a wide variety of intuitions about fitness landscapes, from the null hypothesis of uncorrelated fitness values through explicitly epistatic models to the holey fitness landscapes derived from neutral theory, thus covering all classes of fitness landscapes that are expected to be relevant for real organisms. With the exception of the extreme case of uncorrelated fitness values, which is ruled out by comparison to the empirical data, all models show that fitness landscapes become highly accessible in the biologically relevant limit of large 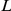: The probability of finding at least one accessible path is an increasing function of 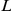 which we conjecture to reach unity for 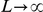, and the expected number of paths grows with 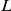 without bounds. The latter feature limits the repeatability of evolutionary trajectories.

In view of the robustness of these properties, we believe that their origin lies in the topological structure of the configuration space: The probability of accessibility of a given path (and thus the relative *fraction* of accessible paths) decreases exponentially with 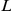, but this is overwhelmed by the combinatorial proliferation of possible paths (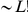), see Eq. (5) for the neutral model and Eq. (8) for the RMF model. As we have imposed severe constraints on the adaptive process by prohibiting the crossing of fitness valleys by double mutations and by only considering shortest paths, our estimate of accessibility is rather conservative. We therefore expect that naturally occurring, genome-wide fitness landscapes should show a very high degree of accessibility as well.

A second general conclusion of our study is that pathway accessibility in epistatic fitness landscapes is subject to large fluctuations, as evidenced by the typical form of the probability distribution 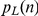 in Figure 2 and Figures S6, S7. For landscape dimensionalities 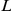 in the range relevant for the available empirical studies, a substantial fraction of landscapes, given by 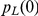, does not possess a single accessible pathway. On the other hand, for all models except the HoC model, the average number of accessible pathways exceeds unity and increases rapidly with increasing 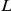. This implies that in those landscapes in which the maximum is accessible at all, it is typically accessible through a large number of pathways. For example, among the 70 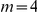 subgraphs of the *A. niger* landscape, half do not contain a single accessible path, but the average number of paths among the graphs with 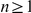 is 4, and two subgraphs display as many as 10 accessible paths.

This observation becomes relevant when applying similar analyses to empirical fitness landscapes based on mutations that are collectively beneficial, such as the examples described in [4], [15], [16]. In these cases the adapted multiple mutant could not have been formed easily by natural selection (alone) unless at least one selectively accessible pathway from the wildtype to the mutant existed. The statistics of such landscapes is therefore biased towards larger accessibility, and a comparison with random models should then be based on the probability distribution 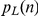 conditioned on 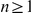. The general question as to whether landscapes formed by combinations of beneficial or deleterious mutations have similar topographical properties can only be answered by further empirical studies.

### The *A. niger* landscape

The analysis of accessible mutational pathways in the empirical *A. niger* data set has allowed us to quantify the amount of sign epistasis in this landscape in terms of model parameters like the roughness scale 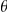 in the RMF model or the number of interacting loci 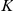 in the 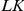 model. Similar to a recent experimental study of viral adaptation [45], we ruled out the null model of a completely uncorrelated fitness landscape. Nevertheless our results suggest that the epistatic interactions in this system are remarkably strong. To put our estimate of 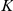 into perspective, we carried out a subgraph analysis of the TEM 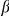-lactamase antibiotic resistance landscape obtained in [3] (Figure S8). In this case the number of loci is 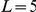, and the comparison of the mean number of accessible paths in subgraphs of sizes 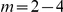 with simulation results for the 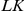 model suggests that 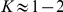, significantly smaller than the estimate 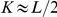 obtained for the *A. niger* landscape. A low value of 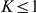 was also found in the analysis of a DNA-protein affinity landscape for the set of all possible 10 base oligomers [46].

Our finding of a high level of intergenic sign epistasis, compared to the examples of intragenic epistasis considered in [3] and [46], contradicts the general expectation that epistatic interactions should be stronger within genes than between genes [15], [16], [47]. Note, however, that the comparisons among the available epistasis data are confounded by differences in the combined fitness of the mutations involved: while the *A. niger* mutations were chosen without a priori knowledge of their (combined) fitness effects, the mutations considered in most studies were known to be collectively beneficial [3], [4], [9], [13], [15], [16], and hence biased against negative epistatic combinations.

### Population dynamics

In the present paper we have focused on the existence of accessible mutational pathways, without explicitly addressing the probability that a given pathway will actually be found under a specific evolutionary scenario. This probability is expected to depend on population parameters, primarily on the mutation supply rate 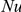, in a complex way. In the SSWM regime characterized by 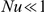 it is straightforward in principle to assign probabilistic weights to mutational pathways in terms of the known transition probabilities of the individual steps [3], [26]. For larger populations additional effects come into play, whose bearing on accessibility and predictability is difficult to assess.

On the one hand, an increase in the mutation supply rate 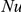 may bias adaptation towards the use of mutations of large beneficial effects, which makes the evolutionary process more deterministic [24] but also more prone to trapping at local fitness maxima [48]. While this reduces the accessibility of the global optimum, at the same time the crossing of fitness valleys becomes more likely due to the fixation of multiple mutations at once [28], which tests mutants for their short-term evolvability [49] and enlarges the set of possible mutational pathways. We plan to address the interplay between landscape structure and population parameters in their effect on pathway accessibility in a future publication.

## Materials and Methods

### Numerical simulations

For the numerical simulations of random landscapes, fitness values were assigned to each of the 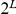 genotypes according to the ensemble to be sampled from (HoC, RMF or 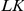 model). The number of paths was then found by a depth-first backtracking algorithm implemented as an iterative subroutine starting at the antipodal genotype and either moving forward, i.e. towards the global fitness maximum, or, if a local maximum is reached, going back to the last genotype encountered before the local maximum. For finding the probability 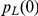 of no accessible paths, the search was ended upon finding the first path, making this search much faster than that for the full distribution of paths and thus enabling us to consider much larger genotype spaces. Results were typically averaged over 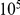 realizations of the random landscape. In analyzing the empirical *A. niger* data, the same routines were used but with the measured fitness values as input instead of fitness values sampled from one of the models.

### Analytic results for the RMF model

It was argued above that both the expected number of accessible paths 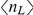 and the probability of no accessible path 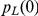 behave fundamentally different for 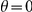 (HoC-model) and the RMF model with strictly positive 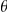, even if 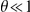. Here we provide additional information on the relation (4) and lend support to the statement that typically 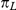, the *probability* of a given path being accessible, decays exponentially in 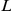. Since by linearity of the expected value 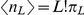, it is sufficient to consider 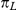 to compute 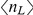.

It was shown in [34] that
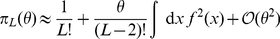
(7)for 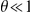, where 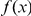 is the probability density of the random fitness contribution 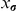. From this form it is clear that the HoC case 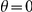 is quite different from the general case 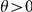. Note that according to (7), 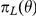 still decays factorially as 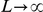. This changes, however, when higher order terms in 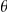 are taken into account.

For the special case when the random fitness contributions are drawn from the Gumbel distribution 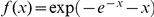, the probability 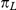 can be computed explicitly for any 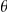 [34]. One obtains the expression
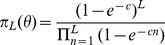
(8)with 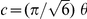. For large 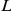, the denominator approaches a constant given by
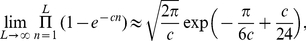
(9)and thus 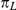 decays exponentially, 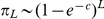. We expect this behavior to be generic for most choices of 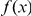.

### Data set

The fitness values constituting the 8-locus empirical data set are presented in Table S1. Here we briefly describe how these values were obtained. A detailed description of the construction and fitness measurement of the *A. niger* strains is given elsewhere [10], [17].

Briefly, *A. niger* is an asexual filamentous fungus with a predominantly haploid life cycle. However, at a low rate haploid nuclei fuse and become diploid; these diploid nuclei are often unstable and generate haploid nuclei by random chromosome segregation. This alternation of ploidy levels resembles the sexual life cycle of haploid organisms and is termed parasexual cycle, since it does not involve two sexes. We exploited the parasexual cycle of *A. niger* to isolate haploid segregants from a diploid strain that originated from a heterokaryon between two strains that were isogenic, except for the presence of eight phenotypic marker mutations in one strain, one on each of its eight chromosomes. These mutations include, in increasing chromosomal order, *fwnA1* (fawn-colored conidiospores), *argH12* (arginine deficiency), *pyrA5* (pyrimidine deficiency), *leuA1* (leucine deficiency), *pheA1* (phenyl-alanine deficiency), *lysD25* (lysine deficiency), *oliC2* (oligomycin resistance), and *crnB12* (chlorate resistance). The wild-type strain only carried a spore-color marker (*olvA1*, causing olive-colored conidiospores) on its first chromosome to allow haploid segregants to be distinguished from the diploid mycelium with black-colored conidiospores. Because these mutations were individually induced with a low dose of UV and combined using the parasexual cycle it was unlikely that the two strains differed at loci other than those of the eight markers.

From the 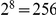 possible haploid segregants, 186 were isolated after forced haploidization of the heterozygous diploid strain on benomyl medium from among 2,500 strains tested. Fitness of all strains was measured with two-fold replication by measuring the linear mycelium growth rate in two perpendicular directions during radial colony growth on supplemented medium that allowed the growth of all strains, and was expressed relative to the mycelium growth rate of the *olvA1* strain with the highest growth rate (see Table S1). As will be explained in the next section, missing genotypes are assigned zero fitness.

### Data analysis

To analyze the data set, first one has to address the problem of missing strains. In the experiments, 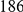 out of 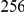 possible strains were found in approximately 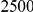 segregants. Assume first that all genotypes are equally likely to be found in the sample. Denoting the number of segregants by 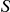, the probability for a given strain to be missed by chance is 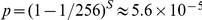. The probability 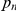 for at most 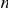 genotypes to have been missed is then given by a Poisson distribution with mean 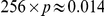. This gives the estimates 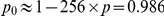 and 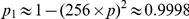. For a more conservative estimate, one may assume that different genotypes have different likelihoods to be found, which are uniformly distributed in the interval 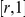 with 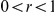. Choosing 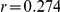 which corresponds to the lowest relative fitness that was observed among the viable genotypes, simulations of this scenario yield 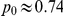 and 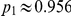. We conclude that it is unlikely that more than one viable genotype has been missed by chance. This justifies the assignment of zero fitness to the missing 70 genotypes.

Next we need to verify that accessibility in the empirical fitness landscape is predominantly determined by sign epistasis among viable genotypes, rather than by the presence of lethals. As described in the main text, we consider subgraphs of the *A. niger* data set containing all combinations of 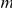 of the eight mutations in total. The set of subgraphs of size 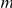 is composed of 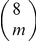 distinct 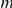-locus landscapes, each of which spans a region in genotype space ranging from the wild type genotype shared by all subgraphs to one particular 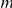-fold mutant. We focus here on the ensembles with 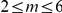.

Key properties of the subgraph ensembles are summarized in Table 1. The first column shows the total number 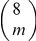 of subgraphs, and the second column shows the number of viable subgraphs (VSG's), defined as subgraphs which contain no non-viable strains. Two of the four VSG's with 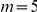 were previously analyzed in [10], and three of the 19 VSG's with 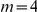 are shown in Figure 1. To assess the impact of lethal genotypes on accessibility, let 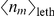 denote the average number of accessible paths per subgraph (averaged across all subgraphs of fixed 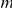) that would be present if *only* lethal states were allowed to block a path and the actual fitness values of viable genotypes were ignored. Similarly, 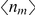 denotes the average number of accessible paths per subgraph for fixed 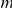 if both mechanisms for blocking are taken into account. Comparison between the two numbers, displayed in the fourth and fifth column of Table 1, shows that the contribution of the lethal mutants to reducing pathway accessibility is relatively minor. For example, for 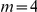 lethals reduce the number of accessible paths from 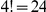 to 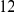, by a factor of 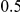, whereas the epistasis among viable genotypes leads to a much more substantial further reduction from 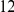 to 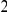, by a factor of 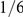; for 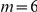 the corresponding factors are 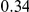 and 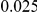. We conclude that pathway accessibility is determined primarily by epistasis among viable genotypes.

Inspection of the VSG's shows that the role of different mutations in causing lethality is strikingly inhomogeneous. In particular, we find that the lysine deficiency mutation *lysD25* is not present in any of the VSG's, whereas the distribution of the other mutations across the VSG's is roughly homogeneous. The *lys* mutation is also strongly overrepresented in the non-viable strains, being present in 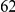 out of 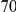 cases. The main features of the set of lethal mutations can be captured in a simple model in which the presence of a mutation 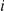 leads to a non-viable strain with probability 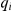, and different mutations interact multiplicatively, such that a strain containing two mutations 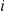 and 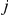 is viable with probability 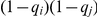. The data for the number of VSG's for different 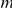 cannot be described assuming the 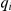 to be the same for all mutations, but a two-parameter model assigning probability 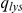 to the 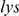 mutation and a common value 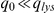 to all others suffices. Simple analysis show that under this model the expected total number of viable strains is 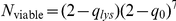, while the total number of viable strains in the subset of strains excluding *lys* is 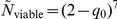. With 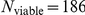 and 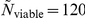 we obtain the estimates 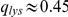 and 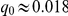. Given that the VSG's do not contain the *lys* mutation, the expected number of VSG's depends only on 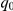, and is given by
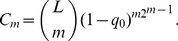
(10)The prediction for the expected number of viable subgraphs is shown in brackets in the third column of Table 1, and is seen to match the data very well. Similarly, the expected number of paths that do not contain any lethal genotypes can be computed analytically, resulting in the expression
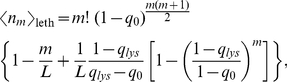
(11)which is shown in brackets in the fourth column of Table 1.

### Resampling procedure

The accessibility of mutational pathways in the *A. niger* data set was analyzed using two different approaches. The first approach is based on a single set of fitness values obtained by averaging the two replicate fitness measurements for each strain; these average fitness values are shown in Table S1. In this approach the fitness assigned to each viable genotype is a normally distributed random variable with the mean given by the average of the two fitness measurements and a common standard deviation 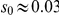 estimated from the mean squared differences between replicate fitness values in the entire data set; the fitness of genotypes identified as non-viable remains zero. Statistical properties of accessible pathways are then computed by averaging over 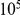 realizations of this resampled landscape ensemble. Empirical data points and error bars shown in Figure 4 represent the mean and standard deviations obtained from the second approach. Results obtained by directly analyzing the mean fitness landscape (first approach) do not differ significantly from those presented here.

## Supporting Information

Figure S1Plot of 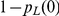 as function of 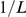 for the HoC model. While the extrapolation to 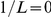 is not straightforward, 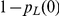 clearly decreases monotonically with a limiting value below 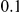.(PDF)

Figure S2Simulation results for the probability of finding no accessible path in the 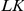 model when the number of non-interacting loci 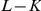 is kept fixed.(PDF)

Figure S3Simulation results for the mean number of accessible paths for the 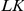 model.(PDF)

Figure S4Simulation results for the probability of finding no shortest connected path between two viable antipodal genotypes for the holey landscape (neutral) model at different viability probabilities 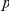. In these simulations the initial genotype and its antipode were constrained to be viable.(PDF)

Figure S5Mean number of accessible paths obtained from subgraph analysis of the *A. niger* landscape (diamonds with error bars) compared to the results of a subgraph analysis of 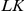 landscapes with 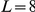, 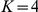 (circles) and 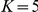 (squares) and 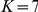 (triangles).(PDF)

Figure S6Distribution of the number of accessible paths in the RMF model with 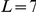. Note that the behavior for the HoC-case 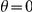 is typical for small values of 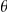 with most of the probabilistic weight on 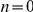. This changes for larger values of 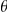, where the probabilistic weight shifts towards many accessible paths. This effect becomes more pronounced as 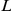 grows.(PDF)

Figure S7Distribution of the number of accessible paths for the LK model with 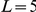 and different values of 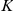. For all 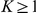, the most likely outcome is 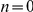. Note the pronounced peaks for 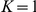, which reflect complex combinatorial correlations among the paths.(PDF)

Figure S8Mean number of accessible paths obtained from subgraph analysis of the TEM 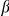-lactamase resistance landscape of Weinreich et al. [3] (squares) compared to the results of a subgraph analysis of 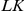 landscapes with 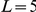, 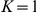 (triangles), 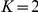 (crosses) and 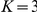 (circles).(PDF)

Table S1Mean fitness 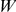 (mycelium growth rate) of the 186 segregants of *A. niger* relative to that of the wildtype strain with the *olv* marker. Presence or absence of marker mutations is indicated with 1 and 0, respectively. Missing genotypes are marked with 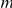.(PDF)
