# Supplementary material for: Evolutionary Accessibility of Mutational Pathways
Source: PLoS Comput Biol. 2011 Aug 18;7(8):e1002134. doi: 10.1371/journal.pcbi.1002134 (PMC3158036; doi:10.1371/journal.pcbi.1002134)
Supplement: Figure S1 — Plot of as function of for the HoC model. While the extrapolation to is not straightforward, clearly decreases monotonically with a limiting value below . (PDF) [file pcbi.1002134.s001.pdf]

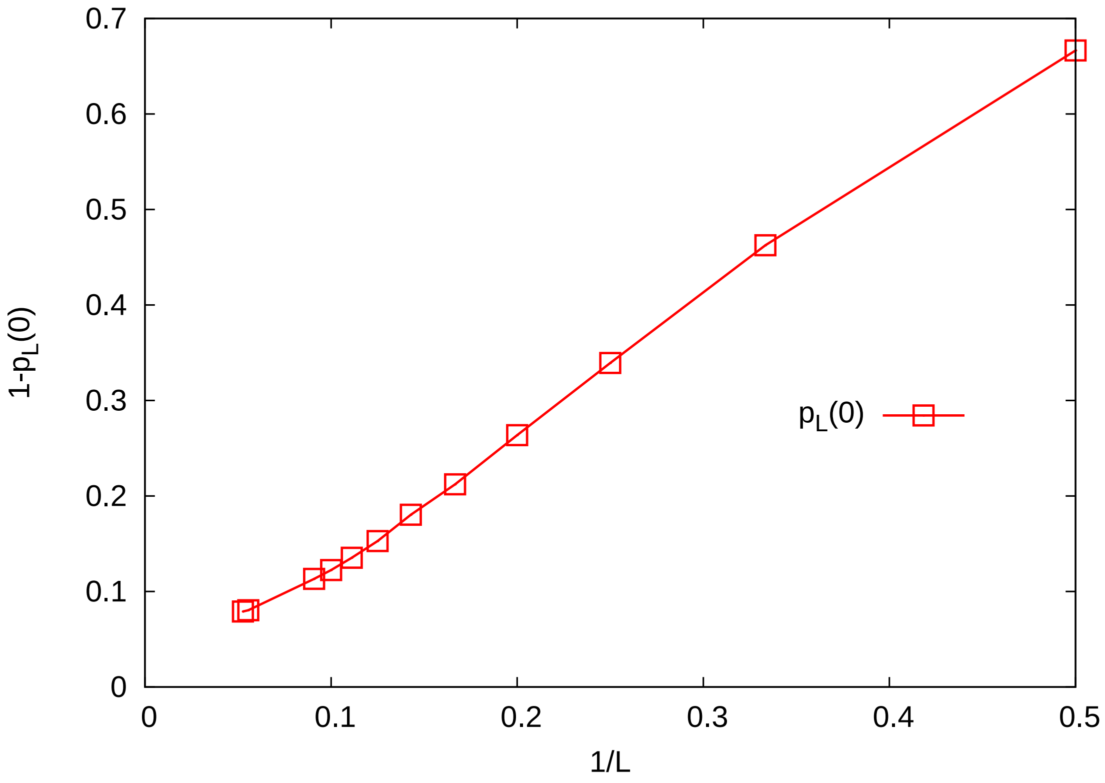

Figure S1: Plot of $1 - p_L(0)$ as function of $1/L$ for the HoC model. While the extrapolation to $1/L = 0$ is not straightforward, $1 - p_L(0)$ clearly decreases monotonically with a limiting value below 0.1.
