# Supplementary material for: Evolutionary Accessibility of Mutational Pathways
Source: PLoS Comput Biol. 2011 Aug 18;7(8):e1002134. doi: 10.1371/journal.pcbi.1002134 (PMC3158036; doi:10.1371/journal.pcbi.1002134)
Supplement: Figure S2 — Simulation results for the probability of finding no accessible path in the model when the number of non-interacting loci is kept fixed. (PDF) [file pcbi.1002134.s002.pdf]

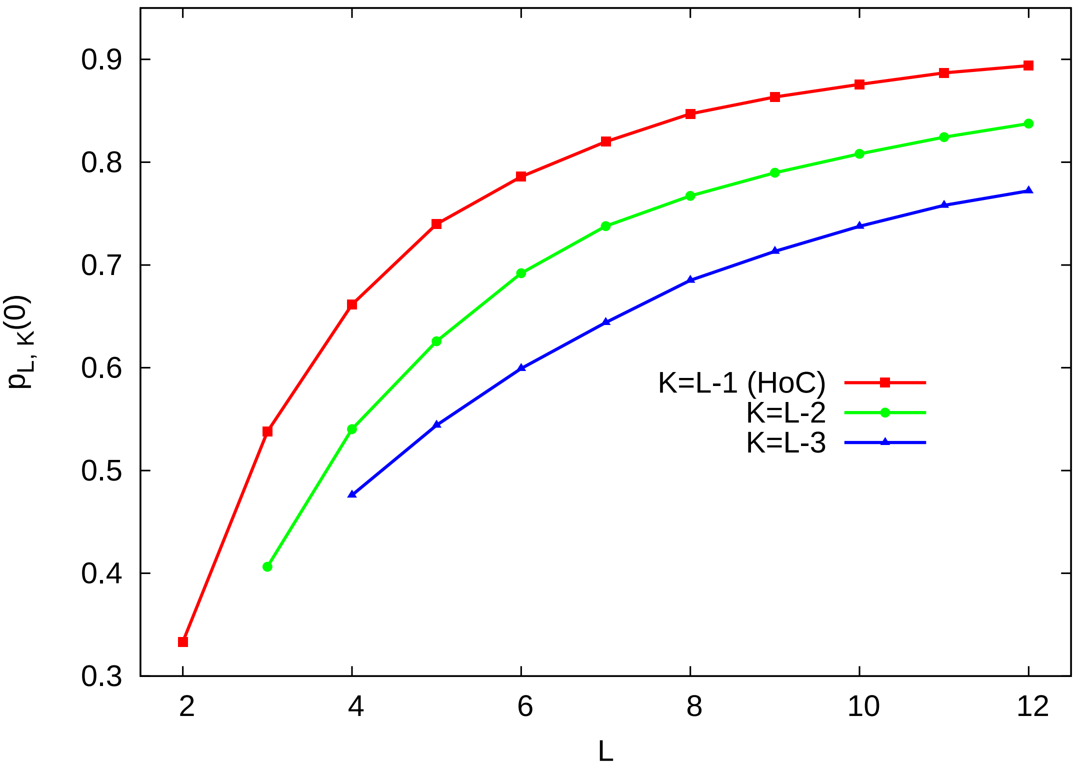

Figure S2: Simulation results for the probability of finding no accessible path in the LK-model when the number of non-interacting loci $L-K$ is kept fixed.
