# Supplementary figures and images for: Evolutionary Accessibility of Mutational Pathways
Source: PLoS Comput Biol. 2011 Aug 18;7(8):e1002134. doi: 10.1371/journal.pcbi.1002134 (PMC3158036; doi:10.1371/journal.pcbi.1002134)

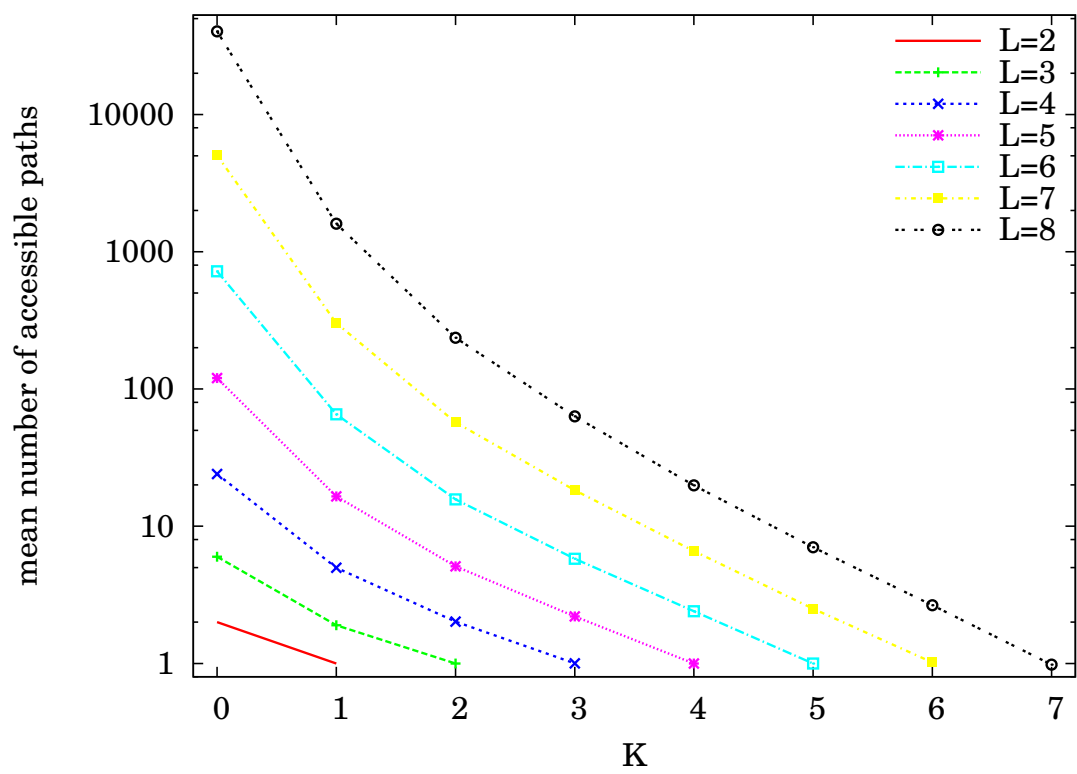

Figure S3: Simulation results for the mean number of accessible paths for the  $LK$  model.

Supplement: Figure S3 — Simulation results for the mean number of accessible paths for the model. (PDF) [file pcbi.1002134.s003.pdf]
