# Supplementary material for: Evolutionary Accessibility of Mutational Pathways
Source: PLoS Comput Biol. 2011 Aug 18;7(8):e1002134. doi: 10.1371/journal.pcbi.1002134 (PMC3158036; doi:10.1371/journal.pcbi.1002134)
Supplement: Figure S4 — Simulation results for the probability of finding no shortest connected path between two viable antipodal genotypes for the holey landscape (neutral) model at different viability probabilities . In these simulations the initial genotype and its antipode were constrained to be viable. (PDF) [file pcbi.1002134.s004.pdf]

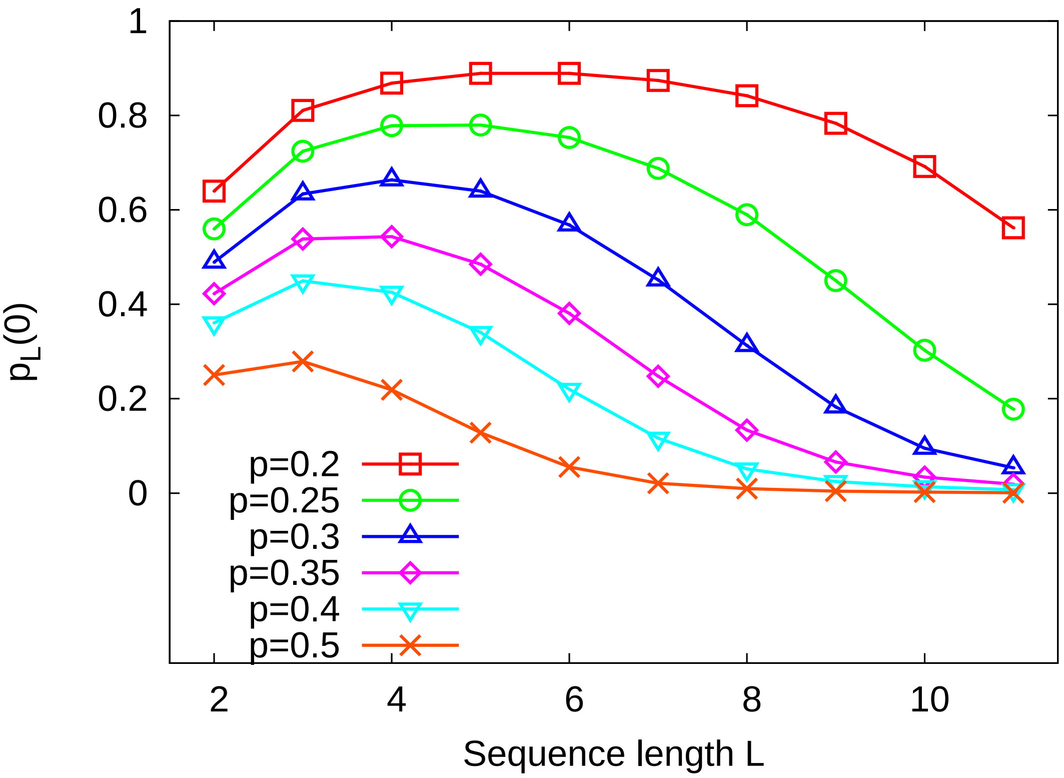

Figure S4: Simulation results for the probability of finding no shortest connected path between two viable antipodal genotypes for the holey landscape (neutral) model at different viability probabilities p. In these simulations the initial genotype and its antipode were constrained to be viable.
