# Supplementary material for: Evolutionary Accessibility of Mutational Pathways
Source: PLoS Comput Biol. 2011 Aug 18;7(8):e1002134. doi: 10.1371/journal.pcbi.1002134 (PMC3158036; doi:10.1371/journal.pcbi.1002134)
Supplement: Figure S5 — Mean number of accessible paths obtained from subgraph analysis of the A. niger landscape (diamonds with error bars) compared to the results of a subgraph analysis of landscapes with , (circles) and (squares) and (triangles). (PDF) [file pcbi.1002134.s005.pdf]

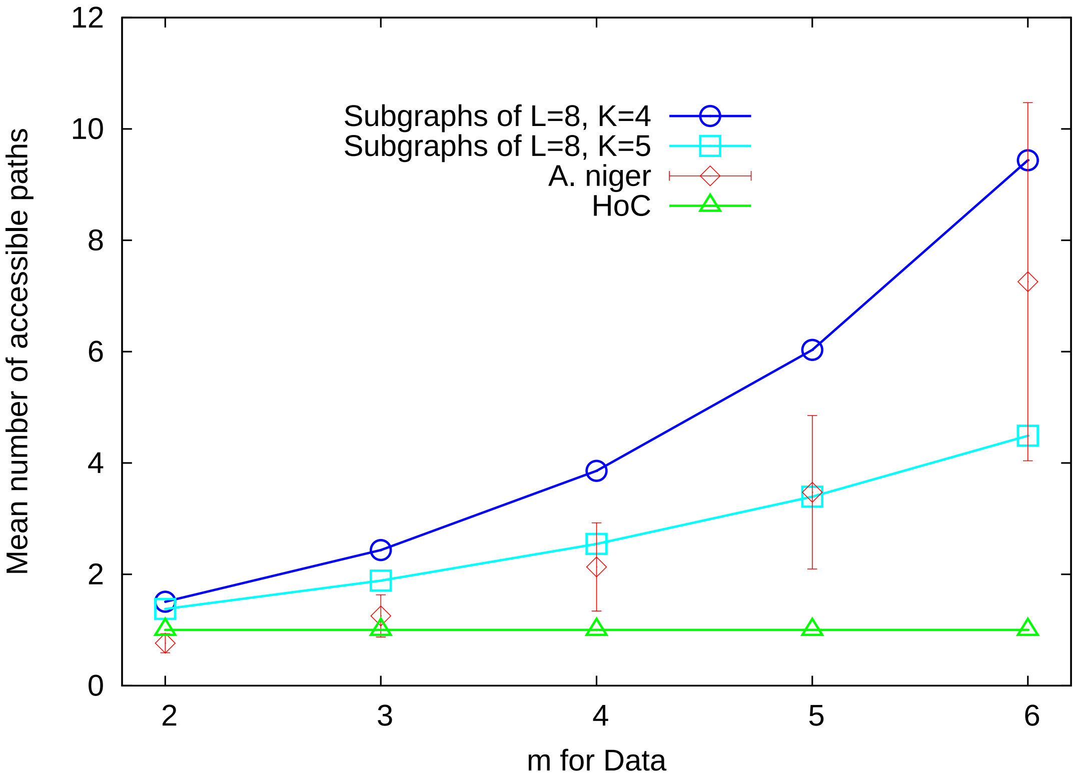

Figure S5: Mean number of accessible paths obtained from subgraph analysis of the *A. niger* landscape (diamonds with error bars) compared to the results of a subgraph analysis of LK-landscapes with $L = 8$, $K = 4$ (circles) and $K = 5$ (squares) and $K = 7$ (triangles).
