# Supplementary material for: Evolutionary Accessibility of Mutational Pathways
Source: PLoS Comput Biol. 2011 Aug 18;7(8):e1002134. doi: 10.1371/journal.pcbi.1002134 (PMC3158036; doi:10.1371/journal.pcbi.1002134)
Supplement: Figure S6 — Distribution of the number of accessible paths in the RMF model with . Note that the behavior for the HoC-case is typical for small values of with most of the probabilistic weight on . This changes for larger values of , where the probabilistic weight shifts towards many accessible paths. This effect becomes more pronounced as grows. (PDF) [file pcbi.1002134.s006.pdf]

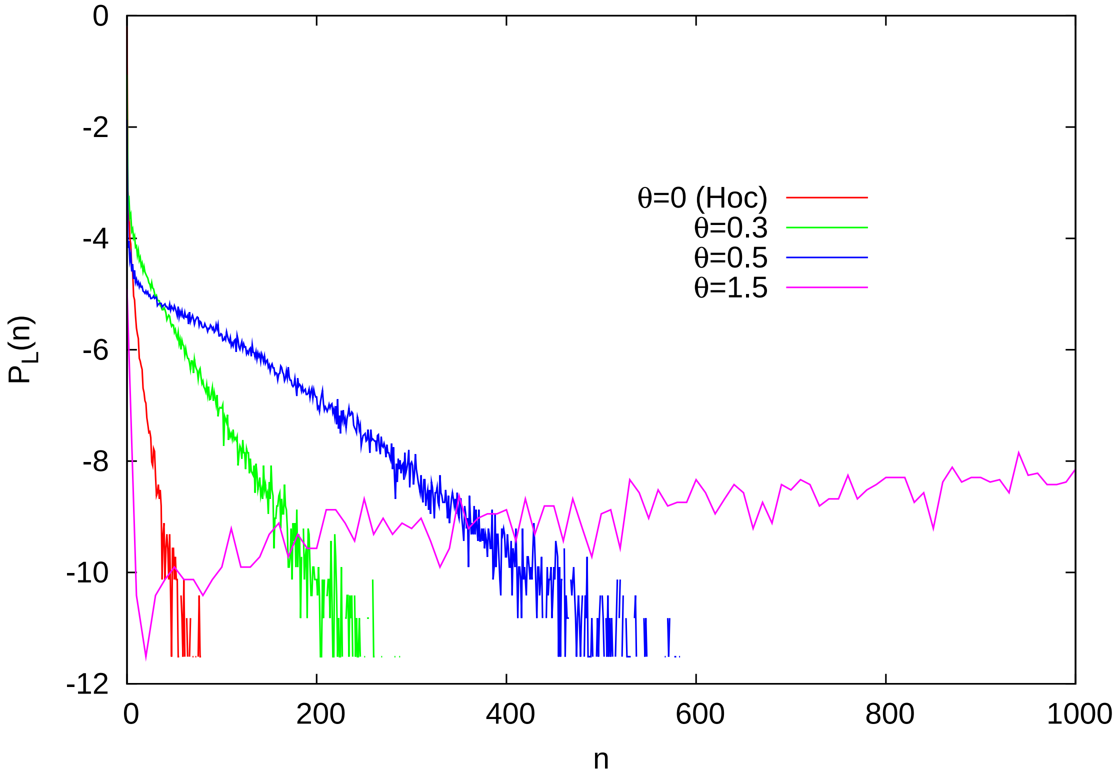

Figure S6: Distribution of the number of accessible paths in the RMF model with $L = 7$. Note that the behavior for the HoC-case $\theta = 0$ is typical for small values of θ with most of the probabilistic weight on $n = 0$. This changes for larger values of θ, where the probabilistic weight shifts towards many accessible paths. This effect becomes more pronounced as L grows.
