# Supplementary material for: Evolutionary Accessibility of Mutational Pathways
Source: PLoS Comput Biol. 2011 Aug 18;7(8):e1002134. doi: 10.1371/journal.pcbi.1002134 (PMC3158036; doi:10.1371/journal.pcbi.1002134)
Supplement: Figure S7 — Distribution of the number of accessible paths for the LK model with and different values of . For all , the most likely outcome is . Note the pronounced peaks for , which reflect complex combinatorial correlations among the paths. (PDF) [file pcbi.1002134.s007.pdf]

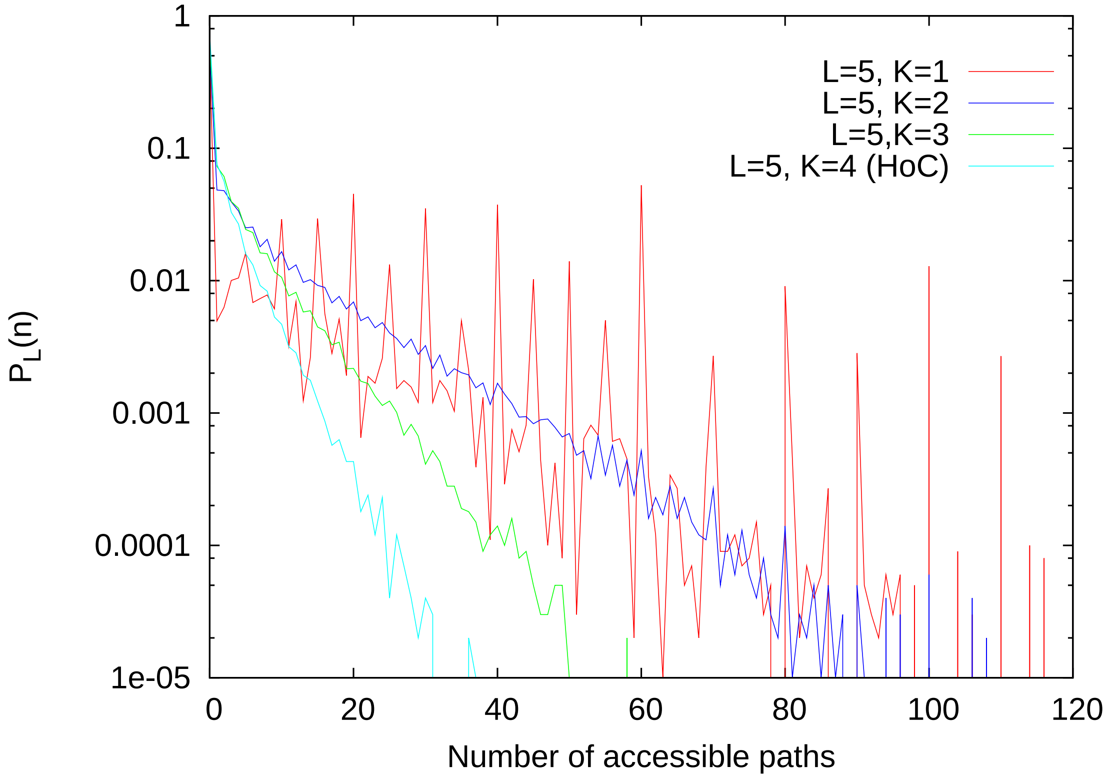

Figure S7: Distribution of the number of accessible paths for the LK-model with $L = 5$ and different values of K. For all $K \geq 1$, the most likely outcome is $n = 0$. Note the pronounced peaks for $K = 1$, which reflect complex combinatorial correlations among the paths.
