# Supplementary material for: Evolutionary Accessibility of Mutational Pathways
Source: PLoS Comput Biol. 2011 Aug 18;7(8):e1002134. doi: 10.1371/journal.pcbi.1002134 (PMC3158036; doi:10.1371/journal.pcbi.1002134)
Supplement: Figure S8 — Mean number of accessible paths obtained from subgraph analysis of the TEM -lactamase resistance landscape of Weinreich et al. [3] (squares) compared to the results of a subgraph analysis of landscapes with , (triangles), (crosses) and (circles). (PDF) [file pcbi.1002134.s008.pdf]

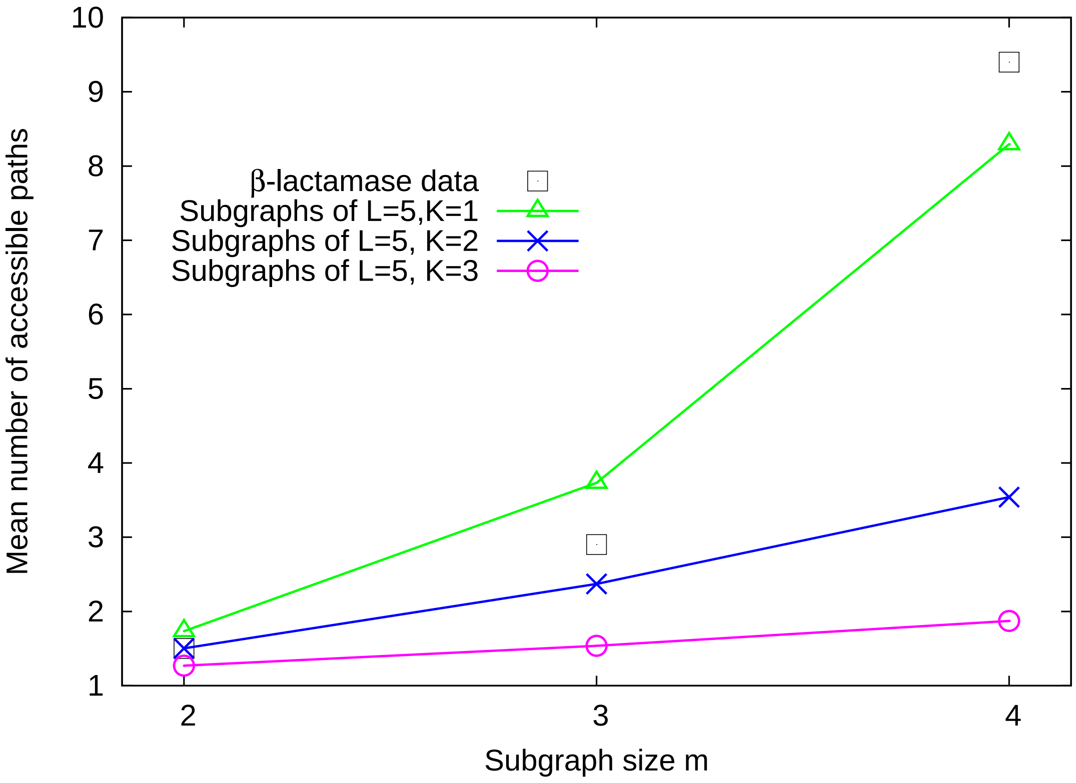

Figure S8: Mean number of accessible paths obtained from subgraph analysis of the TEM1 β-lactamase resistance landscape of Weinreich et al.[3] (squares) compared to the results of a subgraph analysis of LK-landscapes with $L = 5$, $K = 1$ (triangles), $K = 2$ (crosses) and $K = 3$ (circles).
