# Supplementary material for: Evolutionary Accessibility of Mutational Pathways
Source: PLoS Comput Biol. 2011 Aug 18;7(8):e1002134. doi: 10.1371/journal.pcbi.1002134 (PMC3158036; doi:10.1371/journal.pcbi.1002134)
Supplement: Table S1 — Mean fitness (mycelium growth rate) of the 186 segregants of A. niger relative to that of the wildtype strain with the olv marker. Presence or absence of marker mutations is indicated with 1 and 0, respectively. Missing genotypes are marked with . (PDF) [file pcbi.1002134.s009.pdf]

Table S1. Mean fitness *W* (mycelium growth rate) of the 186 segregants of *A. niger* relative to that of the wild-type strain with the *olv* marker. Presence or absence of marker mutations is indicated with 1 and 0, respectively. Missing genotypes are marked with m.

Mutation number	*fwn*	*arg*	*pyr*	*leu*	*phe*	*lys*	*oli*	*crn*	Relative *W*
0	0	0	0	0	0	0	0	0	1.000
1	1	0	0	0	0	0	0	0	0.751
1	0	1	0	0	0	0	0	0	0.773
1	0	0	1	0	0	0	0	0	0.698
1	0	0	0	1	0	0	0	0	0.757
1	0	0	0	0	1	0	0	0	0.826
1	0	0	0	0	0	1	0	0	m
1	0	0	0	0	0	0	1	0	0.598
1	0	0	0	0	0	0	0	1	0.628
2	1	1	0	0	0	0	0	0	0.830
2	1	0	1	0	0	0	0	0	0.765
2	1	0	0	1	0	0	0	0	0.830
2	1	0	0	0	1	0	0	0	0.973
2	1	0	0	0	0	1	0	0	0.747
2	1	0	0	0	0	0	1	0	0.717
2	1	0	0	0	0	0	0	1	0.896
2	0	1	1	0	0	0	0	0	0.748
2	0	1	0	1	0	0	0	0	0.730
2	0	1	0	0	1	0	0	0	0.853
2	0	1	0	0	0	1	0	0	m
2	0	1	0	0	0	0	1	0	0.598
2	0	1	0	0	0	0	0	1	0.762
2	0	0	1	1	0	0	0	0	0.666
2	0	0	1	0	1	0	0	0	0.726
2	0	0	1	0	0	1	0	0	0.722
2	0	0	1	0	0	0	1	0	0.512
2	0	0	1	0	0	0	0	1	0.719
2	0	0	0	1	1	0	0	0	0.731
2	0	0	0	1	0	1	0	0	0.726
2	0	0	0	1	0	0	1	0	0.604
2	0	0	0	1	0	0	0	1	0.672
2	0	0	0	0	1	1	0	0	0.649
2	0	0	0	0	1	0	1	0	0.616
2	0	0	0	0	1	0	0	1	m
2	0	0	0	0	0	1	1	0	m
2	0	0	0	0	0	1	0	1	0.829
2	0	0	0	0	0	0	1	1	0.945
3	1	1	1	0	0	0	0	0	0.783
3	1	1	0	1	0	0	0	0	m

3	1	1	0	0	1	0	0	0	0.750
3	1	1	0	0	0	1	0	0	0.724
3	1	1	0	0	0	0	1	0	0.543
3	1	1	0	0	0	0	0	1	0.814
3	1	0	1	1	0	0	0	0	0.576
3	1	0	1	0	1	0	0	0	0.636
3	1	0	1	0	0	1	0	0	0.768
3	1	0	1	0	0	0	1	0	0.516
3	1	0	1	0	0	0	0	1	0.825
3	1	0	0	1	1	0	0	0	0.735
3	1	0	0	1	0	1	0	0	m
3	1	0	0	1	0	0	1	0	0.522
3	1	0	0	1	0	0	0	1	0.706
3	1	0	0	0	1	1	0	0	0.706
3	1	0	0	0	1	0	1	0	0.468
3	1	0	0	0	1	0	0	1	0.845
3	1	0	0	0	0	1	1	0	m
3	1	0	0	0	0	1	0	1	0.748
3	1	0	0	0	0	0	1	1	0.738
3	0	1	1	1	0	0	0	0	0.667
3	0	1	1	0	1	0	0	0	0.772
3	0	1	1	0	0	1	0	0	m
3	0	1	1	0	0	0	1	0	0.559
3	0	1	1	0	0	0	0	1	0.692
3	0	1	0	1	1	0	0	0	0.887
3	0	1	0	1	0	1	0	0	m
3	0	1	0	1	0	0	1	0	0.562
3	0	1	0	1	0	0	0	1	0.627
3	0	1	0	0	1	1	0	0	m
3	0	1	0	0	1	0	1	0	0.633
3	0	1	0	0	1	0	0	1	0.777
3	0	1	0	0	0	1	1	0	0.457
3	0	1	0	0	0	1	0	1	0.824
3	0	1	0	0	0	0	1	1	0.567
3	0	0	1	1	1	0	0	0	0.736
3	0	0	1	1	0	1	0	0	0.738
3	0	0	1	1	0	0	1	0	0.381
3	0	0	1	1	0	0	0	1	0.657
3	0	0	1	0	1	1	0	0	m
3	0	0	1	0	1	0	1	0	0.524
3	0	0	1	0	1	0	0	1	0.805
3	0	0	1	0	0	1	1	0	m
3	0	0	1	0	0	1	0	1	0.866
3	0	0	1	0	0	0	1	1	0.414
3	0	0	0	1	1	1	0	0	m
3	0	0	0	1	1	0	1	0	0.555

3	0	0	0	1	1	0	0	1	0.655
3	0	0	0	1	0	1	1	0	m
3	0	0	0	1	0	1	0	1	0.795
3	0	0	0	1	0	0	1	1	0.451
3	0	0	0	0	1	1	1	0	m
3	0	0	0	0	1	1	0	1	0.928
3	0	0	0	0	1	0	1	1	m
3	0	0	0	0	0	1	1	1	0.605
4	1	1	1	1	0	0	0	0	0.622
4	1	1	1	0	1	0	0	0	0.735
4	1	1	1	0	0	1	0	0	m
4	1	1	1	0	0	0	1	0	0.523
4	1	1	1	0	0	0	0	1	0.783
4	1	1	0	1	1	0	0	0	0.850
4	1	1	0	1	0	1	0	0	m
4	1	1	0	1	0	0	1	0	0.491
4	1	1	0	1	0	0	0	1	0.700
4	1	1	0	0	1	1	0	0	0.770
4	1	1	0	0	1	0	1	0	m
4	1	1	0	0	1	0	0	1	0.837
4	1	1	0	0	0	1	1	0	m
4	1	1	0	0	0	1	0	1	0.841
4	1	1	0	0	0	0	1	1	0.722
4	1	0	1	1	1	0	0	0	m
4	1	0	1	1	0	1	0	0	m
4	1	0	1	1	0	0	1	0	0.274
4	1	0	1	1	0	0	0	1	0.659
4	1	0	1	0	1	1	0	0	0.675
4	1	0	1	0	1	0	1	0	0.539
4	1	0	1	0	1	0	0	1	0.817
4	1	0	1	0	0	1	1	0	m
4	1	0	1	0	0	1	0	1	0.743
4	1	0	1	0	0	0	1	1	0.640
4	1	0	0	1	1	1	0	0	m
4	1	0	0	1	1	0	1	0	0.643
4	1	0	0	1	1	0	0	1	0.723
4	1	0	0	1	0	1	1	0	m
4	1	0	0	1	0	1	0	1	0.798
4	1	0	0	1	0	0	1	1	0.499
4	1	0	0	0	1	1	1	0	m
4	1	0	0	0	1	1	0	1	0.749
4	1	0	0	0	1	0	1	1	0.603
4	1	0	0	0	0	1	1	1	0.651
4	0	1	1	1	1	0	0	0	0.681
4	0	1	1	1	0	1	0	0	m
4	0	1	1	1	0	0	1	0	0.481

4	0	1	1	1	0	0	0	1	0.730
4	0	1	1	0	1	1	0	0	m
4	0	1	1	0	1	0	1	0	0.442
4	0	1	1	0	1	0	0	1	0.769
4	0	1	1	0	0	1	1	0	m
4	0	1	1	0	0	1	0	1	0.812
4	0	1	1	0	0	0	1	1	0.422
4	0	1	0	1	1	1	0	0	m
4	0	1	0	1	1	0	1	0	0.471
4	0	1	0	1	1	0	0	1	m
4	0	1	0	1	0	1	1	0	m
4	0	1	0	1	0	1	0	1	0.750
4	0	1	0	1	0	0	1	1	0.478
4	0	1	0	0	1	1	1	0	m
4	0	1	0	0	1	1	0	1	0.866
4	0	1	0	0	1	0	1	1	m
4	0	1	0	0	0	1	1	1	m
4	0	0	1	1	1	1	0	0	m
4	0	0	1	1	1	0	1	0	0.410
4	0	0	1	1	1	0	0	1	0.702
4	0	0	1	1	0	1	1	0	m
4	0	0	1	1	0	1	0	1	0.793
4	0	0	1	1	0	0	1	1	0.414
4	0	0	1	0	1	1	1	0	m
4	0	0	1	0	1	1	0	1	0.847
4	0	0	1	0	1	0	1	1	0.525
4	0	0	1	0	0	1	1	1	0.659
4	0	0	0	1	1	1	1	0	m
4	0	0	0	1	1	1	0	1	0.767
4	0	0	0	1	1	0	1	1	0.453
4	0	0	0	1	0	1	1	1	0.462
4	0	0	0	0	1	1	1	1	m
5	1	1	1	1	1	0	0	0	0.605
5	1	1	1	1	0	1	0	0	m
5	1	1	1	1	0	0	1	0	0.430
5	1	1	1	1	0	0	0	1	0.686
5	1	1	1	0	1	1	0	0	0.639
5	1	1	1	0	1	0	1	0	0.560
5	1	1	1	0	1	0	0	1	0.707
5	1	1	1	0	0	1	1	0	m
5	1	1	1	0	0	1	0	1	0.794
5	1	1	1	0	0	0	1	1	0.637
5	1	1	0	1	1	1	0	0	0.727
5	1	1	0	1	1	0	1	0	0.604
5	1	1	0	1	1	0	0	1	0.722
5	1	1	0	1	0	1	1	0	m

5	1	1	0	1	0	1	0	1	0.611
5	1	1	0	1	0	0	1	1	0.617
5	1	1	0	0	1	1	1	0	m
5	1	1	0	0	1	1	0	1	0.824
5	1	1	0	0	1	0	1	1	0.638
5	1	1	0	0	0	1	1	1	m
5	1	0	1	1	1	1	0	0	m
5	1	0	1	1	1	0	1	0	0.337
5	1	0	1	1	1	0	0	1	0.647
5	1	0	1	1	0	1	1	0	m
5	1	0	1	1	0	1	0	1	0.701
5	1	0	1	1	0	0	1	1	0.409
5	1	0	1	0	1	1	1	0	m
5	1	0	1	0	1	1	0	1	0.928
5	1	0	1	0	1	0	1	1	0.446
5	1	0	1	0	0	1	1	1	0.619
5	1	0	0	1	1	1	1	0	m
5	1	0	0	1	1	1	0	1	0.720
5	1	0	0	1	1	0	1	1	0.537
5	1	0	0	1	0	1	1	1	m
5	1	0	0	0	1	1	1	1	0.615
5	0	1	1	1	1	1	0	0	m
5	0	1	1	1	1	0	1	0	0.387
5	0	1	1	1	1	0	0	1	0.669
5	0	1	1	1	0	1	1	0	m
5	0	1	1	1	0	1	0	1	0.779
5	0	1	1	1	0	0	1	1	0.416
5	0	1	1	0	1	1	1	0	m
5	0	1	1	0	1	1	0	1	0.827
5	0	1	1	0	1	0	1	1	0.562
5	0	1	1	0	0	1	1	1	0.618
5	0	1	0	1	1	1	1	0	m
5	0	1	0	1	1	1	0	1	0.740
5	0	1	0	1	1	0	1	1	0.469
5	0	1	0	1	0	1	1	1	0.538
5	0	1	0	0	1	1	1	1	0.614
5	0	0	1	1	1	1	1	0	m
5	0	0	1	1	1	1	0	1	0.619
5	0	0	1	1	1	0	1	1	0.414
5	0	0	1	1	0	1	1	1	0.517
5	0	0	1	0	1	1	1	1	0.645
5	0	0	0	1	1	1	1	1	0.454
6	1	1	1	1	1	1	0	0	0.700
6	1	1	1	1	1	0	1	0	0.344
6	1	1	1	1	1	0	0	1	0.691
6	1	1	1	1	0	1	1	0	m

6	1	1	1	1	0	1	0	1	0.662
6	1	1	1	1	0	0	1	1	0.443
6	1	1	1	0	1	1	1	0	m
6	1	1	1	0	1	1	0	1	0.730
6	1	1	1	0	1	0	1	1	0.612
6	1	1	1	0	0	1	1	1	0.629
6	1	1	0	1	1	1	1	0	0.318
6	1	1	0	1	1	1	0	1	0.841
6	1	1	0	1	1	0	1	1	m
6	1	1	0	1	0	1	1	1	m
6	1	1	0	0	1	1	1	1	0.609
6	1	0	1	1	1	1	1	0	m
6	1	0	1	1	1	1	0	1	0.670
6	1	0	1	1	1	0	1	1	0.545
6	1	0	1	1	0	1	1	1	m
6	1	0	1	0	1	1	1	1	0.479
6	1	0	0	1	1	1	1	1	0.475
6	0	1	1	1	1	1	1	0	m
6	0	1	1	1	1	1	0	1	0.790
6	0	1	1	1	1	0	1	1	0.470
6	0	1	1	1	0	1	1	1	m
6	0	1	1	0	1	1	1	1	m
6	0	1	0	1	1	1	1	1	0.514
6	0	0	1	1	1	1	1	1	m
7	0	1	1	1	1	1	1	1	m
7	1	0	1	1	1	1	1	1	m
7	1	1	0	1	1	1	1	1	0.456
7	1	1	1	0	1	1	1	1	0.643
7	1	1	1	1	0	1	1	1	0.446
7	1	1	1	1	1	0	1	1	0.448
7	1	1	1	1	1	1	0	1	m
7	1	1	1	1	1	1	1	0	m
8	1	1	1	1	1	1	1	1	m
